# Existence of HbF Enhancer Haplotypes at* HBS1L-MYB* Intergenic Region in Transfusion-Dependent Saudi *β*-Thalassemia Patients

**DOI:** 10.1155/2017/1972429

**Published:** 2017-02-09

**Authors:** Cyril Cyrus, Chittibabu Vatte, J. Francis Borgio, Abdullah Al-Rubaish, Shahanas Chathoth, Zaki A. Nasserullah, Sana Al Jarrash, Ahmed Sulaiman, Hatem Qutub, Hassan Alsaleem, Alhusain J. Alzahrani, Martin H. Steinberg, Amein K. Al Ali

**Affiliations:** ^1^Department of Genetic Research, Institute for Research and Medical Consultation, University of Dammam, Dammam, Saudi Arabia; ^2^King Fahd Hospital of the University, University of Dammam, Dammam, Saudi Arabia; ^3^Maternity and Children's Hospital, Dammam, Saudi Arabia; ^4^Al-Omran Scientific Chair for Hematological Diseases, King Faisal University, Maternity and Children's Hospital, Dammam, Saudi Arabia; ^5^College of Applied Medical Sciences, King Saud University, Riyadh, Saudi Arabia; ^6^Center of Excellence in Sickle Cell Disease, Department of Medicine, Boston University School of Medicine, Boston, MA, USA

## Abstract

*Background and Objectives*. *β*-Thalassemia and sickle cell disease are genetic disorders characterized by reduced and abnormal *β*-globin chain production, respectively. The elevation of fetal hemoglobin (HbF) can ameliorate the severity of these disorders. In sickle cell disease patients, the HbF level elevation is associated with three quantitative trait loci (QTLs),* BCL11A*,* HBG*2 promoter, and* HBS1L-MYB *intergenic region. This study elucidates the existence of the variants in these three QTLs to determine their association with HbF levels of transfusion-dependent Saudi *β*-thalassemia patients.* Materials and Methods*. A total of 174 transfusion-dependent *β*-thalassemia patients and 164 healthy controls from Eastern Province of Saudi Arabia were genotyped for fourteen single nucleotide polymorphisms (SNPs) from the three QTL regions using TaqMan assay on real-time PCR.* Results*. Genotype analysis revealed that six alleles of* HBS1L-MYB *QTL (rs9376090C *p* = 0.0009, rs9399137C *p* = 0.008, rs4895441G *p* = 0.004, rs9389269C *p* = 0.008, rs9402686A *p* = 0.008, and rs9494142C *p* = 0.002) were predominantly associated with *β*-thalassemia. In addition, haplotype analysis revealed that haplotypes of* HBS1L-MYB *(GCCGCAC *p* = 0.022) and* HBG*2 (GTT *p* = 0.009) were also predominantly associated with *β*-thalassemia. Furthermore, the* HBS1L-MYB *region also exhibited association with the high HbF cohort.* Conclusion*. The stimulation of HbF gene expression may provide alternative therapies for the amelioration of the disease severity of *β*-thalassemia.

## 1. Introduction

The most common hereditary hemolytic anemias are *β*-thalassemia and sickle cell disease (SCD). *β*-Thalassemia is characterized by absence or reduction of *β*-globin chain synthesis, while SCD is characterized by the production of abnormal *β*-globin chain. However, the pathophysiology of each disorder is different [1]. Both SCD and *β*-thalassemia are prevalent in the Eastern Province of Saudi Arabia and are characterized by a wide range of phenotypic heterogeneity [2–7]. Fetal hemoglobin (HbF; *α*2*γ*2) is a major genetic modifier of disease severity in SCD [8]. Elevated HbF can ameliorate the clinical and hematologic severity of the disease and persistently elevated HbF partially compensates for the lack of HbA in *β*-thalassemia and also decreases *α*/*β* chain imbalance and the consequent toxicity of unpaired *α*-globin chains [9]. In Saudi *β*-thalassemia patients, a highly elevated level of HbF, ranging from 40 to 98%, has been observed [10].

Understanding the regulation of* HBG* (*γ*-globin gene) expression is of both biological and clinical relevance [9]. A section of DNA locus that correlates with phenotypic variation is known as quantitative trait locus (QTL). The first identified QTL associated with an elevated HbF level was the −158 C>T,* XmnI *site (rs7482144), at the 5′ to* HBG*2 [11]. In the same region, that is, SNP rs5006884 in olfactory receptor (OR) genes (*OR51B5* and* OR51B6*), upstream of the *β*-globin gene cluster has been reported to be associated with elevated HbF level in several populations. The rs2071348 in the *β*-globin locus is also in tight linkage disequilibrium with rs7482144* (HBG*2) and is associated with elevated HbF [12]. Two other QTLs, located in the* HBS1L-MYB* intergenic region and in the* BCL11A* gene, are either directly involved in HbF gene silencing in adult life or in cell proliferation and differentiation [9, 13–15].* BCL11A* (2p16.1),* HBS1L-MYB *(6q23.3) and* HBG*2 promoter regions account for approximately 10–50% of HbF variation depending on the population studied, with the remaining variance in HbF level unaccounted for, indicating that additional loci are involved [16]. More recently, a polymorphism in intron 9 of* ANTXR1*, a type 1 transmembrane protein and receptor for anthrax toxin, was found to be associated with elevated HbF in Saudi patients with the AI haplotype [17]. Therefore, the objective of this study was to determine the existence of known HbF enhancer loci,* BCL11A*,* HBG*, and* HBS1L-MYB *polymorphisms, and their haplotypes, in transfusion-dependent Saudi *β*-thalassemia patients.

## 2. Materials and Methods

This is a case control study conducted on 174 transfusion-dependent *β*-thalassemia patients (age range 2 to 18 years; 93 males and 81 females) and 164 age and sex matched healthy controls from the Eastern Province of Saudi Arabia. All *β*-thalassemia patients attending three major hospitals in the Eastern Province, namely, King Fahd Hospital of the University, Dammam; Maternity and Children's Hospital, Dammam; and King Fahd Hospital, Al-Ahssa, were requested to participate in the study. All the patients included in this study were clinically diagnosed with *β*-thalassemia major. In addition, the *β*-thalassemia mutations in the majority of these patients have been identified and reported previously [2, 7]. The HbF levels reported in this manuscript represent the first baseline measurement for these patients, who are transfused regularly every two to three weeks. The patients' mean hemoglobin was maintained at approximately above 7.0 g/dL. All the controls were randomly selected from the general population with no history or family history of *β*-thalassemia or SCD and from the same area.

This study was approved by the Ethical Committee of the University of Dammam in accordance with the 1964 Helsinki Declaration and its later amendments. Signed written informed consent was obtained from all participants. Blood samples were collected in EDTA vacutainers and DNA was extracted using blood minikit (Qiagen, GmbH, Hilden, Germany). HbF levels were determined using Bio-Rad Variant II (Variant II *β*-Thalassemia Short Program Recorder Kit, Hercules, CA 94547, USA). The patient cohort was subgrouped based on HbF level, with 106 patients having a HbF level > 40% and 68 patients having a HbF level < 40%. SNP genotyping was carried out by nuclease allelic discrimination assay with target-specific forward and reverse primers along with TaqMan probes (Applied Biosystems, Foster City, California, USA) labeled with VIC and FAM for each allele on the ABI 7500 real-time PCR system (Applied Biosystems, Foster City, California, USA) according to the manufacturer's instructions. Fourteen SNPs, namely, rs2071348, rs7482144, and rs5006884 (*HBG2* promoter region), rs766432, rs11886868, rs4671393, and rs7557939 (*BCL11A* region), and rs28384513, rs9376090, rs9399137, rs4895441, rs9389269, rs9402686, and rs9494142 (*HBS1L-MYB *region), were studied. All the SNPs were tested for Hardy-Weinberg equilibrium (HWE). Chi square and odds ratio was determined by SPSS version 19 to evaluate allele association. Linkage disequilibrium (LD) test was carried out using HaploView 4.2 software program to identify the nonrandom association of these 14 SNPs. Haplotype blocks were constructed using HaploView 4.2 program [18]. Haplotypes associated with *β*-thalassemia were inferred based on the partition-ligation approach through EM algorithm. A *p* value below 0.05 was considered significant for all statistical analyses.

## 3. Results

The mean of hemoglobin level, mean corpuscular volume, ferritin, and iron of the 174 transfusion-dependent *β*-thalassemia patients were 7.89 ± 1.66 g/dL, 68.93 ± 9.31 fl, 3222 ± 2396 *μ*g/L, and 81.05 ± 70.39 *μ*mol/L, respectively. The range of HbF in patients' cohort was 1.4 to 99.4% (83.77 ± 23.78%). Only five patients were found to have HbF level below 10% (1.4%  *n* = 1; 5.9%  *n* = 1; 8.1%  *n* = 1 and 9.8%  *n* = 2). All other patients in the low HbF cohort had a value HbF of between 10–39.1%. From the total patient group, the HbF pretransfusion level was >40% in 106 patients. All patients are treated with Deferasirox (Exjade, Novartis Pharmaceuticals Ltd., UK) an orally administered iron chelation agent. From the study group, 53 patients had splenomegaly, 20 undergone splenectomy, and 18 had bone abnormalities.

The independent segregation genotype for all the SNPs in the control group was in agreement with the Hardy-Weinberg equilibrium. Standard allelic association analysis of the 14 SNPs tested in the patient cohort showed that only six SNPs in the* HBS1L-MYB *region, namely, rs9376090, rs9399137, rs4895441, rs9389269, rs9402686, and rs9494142, were significantly associated with *β*-thalassemia. There were no significant differences in allele frequencies of SNPs in the* HBG*2 promoter region and* BCL11A* region between the *β*-thalassemia and control groups (Table 1). However, when the patients were subgrouped into those who had HbF > 40% (106 patients) and those who had HbF < 40% (68 patients), the group with HbF > 40% showed a significant association with the six *β*-thalassemia associated SNPs, in addition to two other SNPs, namely, rs7557939 (OR = 1.54, *p* = 0.013) and rs11886868 (OR = 1.47, *p* = 0.029) on* BCL11A* locus. The subgroup with HbF <40% showed only rs2071348* on HBG2* promoter region to be associated with *β*-thalassemia (Table S1 in Supplementary Material available online at https://doi.org/10.1155/2017/1972429).

**Figure 1:**
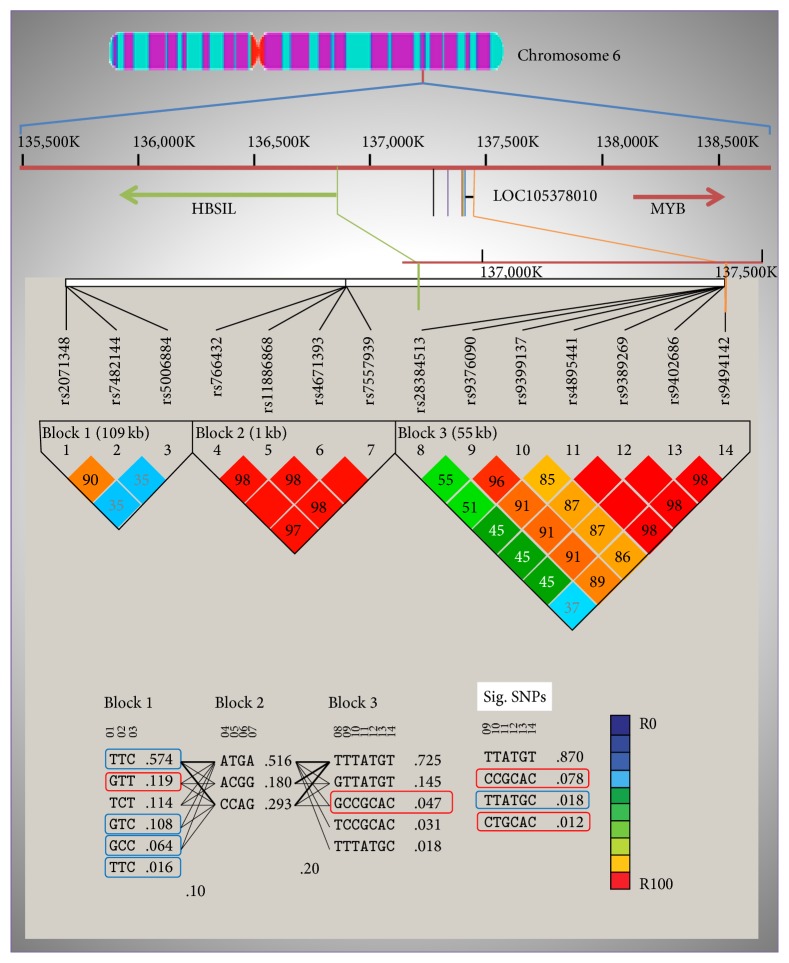
Linkage disequilibrium (LD) analysis patterns between 3* HBG2,* 4* BCL11A*, and 7* HBS1L-MYB* SNPs compared in thalassemia patients against control cohort. The figure illustrates the chromosome 6 loci inhabiting the seven* HBS1L-MYB *interregion SNPs outlining the chromosomal location, SNP ID, and the positions that were genotyped in this study. HaploView output of LD across 14 SNPs from the genotyping data in Saudi population. The pairwise correlation between the SNPs was measured as *r*^2^ and shown (×100) in each diamond. Enhancer haplotypes are in red boxes and diminisher haplotypes are in blue boxes. Coordinates are according to the NCBI build dbSNP 144* Homo sapiens* annotation release 107 (reference sequence NT_025741.16). Sig. SNPs: haplotypes of significant SNPs.

The predominant haplotypes in the whole *β*-thalassemia cohort consisted of GTT and TCC in the* HBG*2 promoter region and GCCGCAC (*p* = 0.022; *χ*^2^ = 5.257) in the* HBS1L-MYB *region (Figure 1). The CCGCAC (rs9376090C, rs9399137C, rs4895441G, rs9389269C, rs9402686A, and rs9494142C) haplotype pattern was the most significant HbF enhancer haplotype (*p* = 0.005; *χ*^2^ = 7.739) followed by CTGCAC (*p* = 0.04; *χ*^2^ = 4.181) (Table 2). The predominant haplotypes in the subgroup with HbF > 40% consisted of GTT in the* HBG*2 promoter region, ATGA in* BCL11A*, and GCCGCAC in the* HBS1L-MYB *region. The predominant haplotypes in the subgroup with HbF < 40% consisted of TCC in the* HBG*2 promoter region (Table S2).

## 4. Discussion

Human erythroid progenitor based functional studies revealed that reduced transcription factor bindings, which could affect long-range interactions with* MYB* due to common variants within the intergenic region* (HBS1L-MYB)*, result in reduced* MYB* expression leading to elevated HbF levels [19]. In addition, common variants have been identified to be associated with elevated HbF in the* BCL11A* region and* HBG*2 promotor region in SCD [20]. The stimulation of HbF expression may provide alternative therapies for the amelioration of disease severity in *β*-thalassemia and SCD [21]. Increased knowledge and understanding of the genetics of HbF regulation supports the development of innovative therapeutic targets, including the development of novel drug therapies.

To the best of our knowledge, this is the first study reporting the influence of 14 genetic markers spanning the three important QTLs, namely,* HBG2* promoter,* BCL11A*, and* HBS1L-MYB *regions in *β*-thalassemia major patients. In this study, we examined selected SNPs in the* BCL11A*,* HBG2*, and* HBS1L-MYB *loci on chromosomes 11p15.4, 2p16.1, and 6q23.3, respectively, in Saudi *β*-thalassemia patients from the Eastern Province to determine their association with HbF levels. The selection of the SNPs was based on recently published studies, which reported that these genetic variants were most strongly associated with increased HbF levels in SCD and *β*-thalassemia intermedia type of patients [9, 20, 22–28].

Six of the 14 SNPs in the* HBS1L-MYB *region showed a strong association with *β*-thalassemia. This is consistent with previous reports from European, Chinese and African *β*-thalassemia intermedia and SCD patients [9, 13, 20, 22, 23, 25, 26, 28]. However, two of these SNPs (rs4895441 and rs93991370) did not show an association in SCD patients from the South-Western Province of Saudi Arabia [3]. It has to be noted that SCD in the Eastern Province carries the Arab-Indian haplotype, while in the South-Western Province, SCD patients carry the Benin haplotype [3].

The effects of* BCL11A* QTL on HbF levels have been reported in *β*-thalassemia intermedia in different populations [29, 30]. In the present study, two SNPs, namely, rs7557939 and rs11886868, were found to be associated with *β*-thalassemia in patients with HbF level > 40%. The other SNPs in the same region showed a lack of association with *β*-thalassemia, in contrast to other studies conducted on Chinese and Portuguese populations [28, 31]. The lack of association with the transfusion-dependent *β*-thalassemia major and association with the Hb E/*β*-thalassemia cases [31] and beta-thalassemia carriers [28] suggests that rs4671393, rs7557939, and rs11886868 are HbF enhancer SNPs in *β*-thalassemia intermedia.

It has been reported that the* XmnI*  ^G^*γ*-158(C→T) polymorphism (rs7482144) of* HBG2* was associated with increased production of ^G^*γ* globin, and hence HbF can influence the heterogeneity of both blood transfusion-dependent and transfusion-independent *β*-thalassemia patients [32–37]. Although this SNP was reported to be associated with *β*-thalassemia in a number of populations, in our cohort this association is lacking [24, 38]. Moreover, other SNPs (rs2071348 and rs5006884) in the* HBG*2 promoter region were shown to lack an association with *β*-thalassemia in our cohort.

Haplotype analysis showed that CCGCAC in the* HBS1L-MYB *region is strongly associated with *β*-thalassemia in our cohort (*χ*^2^ = 7.739; *p* = 0.005), while in the* HBG*2 region the haplotypes GTT (*χ*^2^ = 6.767; *p* = 0.009) and TCC (*χ*^2^ = 5.652; *p* = 0.017) showed a strong association with *β*-thalassemia. Paucity of literature on the GTT haplotypes among *β*-thalassemia prevents the comparison of their effect.

The haplotype analysis of present and previous studies of the SNPs from the three tested regions (*HBG*2 locus,* BCL11A*, and the* HBS1L-MYB *interregion) showed stronger association with elevated HbF level than single SNPs taken individually [15, 39]. Moreover, it has been shown that the distribution of* BCL11A* enhancer haplotypes showed significant differences based on geographical origin accounting for the HbF level deviation [40]. Interestingly, the ATGA haplotype formed from the four SNPs rs766432, rs11886868, rs4671393, and rs7557939, though it lacked an association with *β*-thalassemia major. However, this haplotype was found to be associated with HbF in the subgroup of patients with HbF > 40%. This haplotype has been previously reported to be associated with elevated HbF in Saudi SCD patients from the Eastern Province [40].

## 5. Conclusion 

The stimulation of HbF expression may provide alternative therapies for the amelioration of the disease severity of *β*-thalassemia and SCD. Furthermore, increasing knowledge and understanding of the genetics of HbF regulation will support the development of innovative therapeutic targets, including the development of novel drug therapies. Therefore, our study provided valuable insights on the elements that influence elevated HbF levels in *β*-thalassemia.

## Supplementary Material

Table S1: Allelic association of 14 SNPs related to BCL11A, HBS1L-MYB, and HBG2 promoter region in patients with β-thalassemia and elevated HbF levels. Table S2: Frequency of haplotypes of SNPs in HBG2, BCL11A, and HBS1L-MYB compared between High HbF and Control cohorts.

## Figures and Tables

**Table 1:** Allelic association of 14 SNPs related to *BCL11A*, *HBS1L-MYB*, and *HBG2* promoter region in patients with *β*-thalassemia and controls.

SNP ID	Candidate gene	Chromosome position	Alleles (EA/OA)	Case versus control		
				*χ* ^2^	Odds ratio (95% CI)	*p* value
rs2071348	*HBG2* region	11:5242916	T/G	1.184	0.832 (0.598–1.159)	0.277
rs7482144	*HBG2* region	11:5254939	C/T	0.498	0.881 (0.620–1.252)	0.480
rs5006884	*HBG2* region	11:5352021	T/C	1.222	0.818 (0.577–1.168)	0.269
rs766432	*BCL11A*	2:60492835	A/C	0	0.999 (0.719–1.388)	0.997
rs11886868	*BCL11A*	2:60493111	T/C	1.637	0.821 (0.607–1.111)	0.201
rs4671393	*BCL11A*	2:60493816	G/A	0.006	0.987 (0.709–1.375)	0.940
rs7557939	*BCL11A*	2:60494212	A/G	2.055	0.802 (0.592–1.085)	0.152
rs28384513	*HBS1L-MYB*	6:135055071	G/T	2.496	0.741 (0.511–1.075)	0.114
rs9376090	*HBS1L-MYB*	6:135090090	C/T	11.053	0.406 (0.235–0.700)	0.0009^*∗*^
rs9399137	*HBS1L-MYB*	6:135097880	C/T	7.228	0.473 (0.271–0.824)	0.008^*∗*^
rs4895441	*HBS1L-MYB*	6:135105435	G/A	8.785	0.449 (0.262–0.771)	0.004^*∗*^
rs9389269	*HBS1L-MYB*	6:135106021	C/T	7.096	0.495 (0.293–0.837)	0.008^*∗*^
rs9402686	*HBS1L-MYB*	6:135106679	A/G	7.096	0.495 (0.293–0.837)	0.008^*∗*^
rs9494142	*HBS1L-MYB*	6:135110502	C/T	9.936	0.459 (0.280–0.751)	0.002^*∗*^

^*∗*^Significant association *p* values (*p* < 0.05) for the allelic model. EA: effect allele tested for association; OA: other allele; *p: p* value unadjusted; chromosome position as per GRCh38.p2 Assembly.

**Table 2:** Frequency of haplotypes of SNPs in *HBG2, BCL11A*, and *HBS1L-MYB *compared between patients with *β*-thalassemia and control cohorts.

Block	Candidate gene	Haplotype	Case versus control			
			Overall frequency	Case; control frequencies	*χ* ^2^	*p* value
1	*HBG2* region	TCC	0.574	0.618, 0.528	5.652	0.0174^*ϕ*^
	*HBG2* region	**GTT**	0.119	0.150, 0.086	6.767	0.0093^*∗*^
	*HBG2* region	TCT	0.114	0.103, 0.126	0.907	0.3409
	*HBG2* region	GTC	0.108	0.079, 0.138	6.136	0.0132^*ϕ*^
	*HBG2* region	GCC	0.064	0.044, 0.085	4.858	0.0275^*ϕ*^
	*HBG2* region	TTC	0.016	0.003, 0.029	7.306	0.0069^*ϕ*^

2	*BCL11A*	ATGA	0.516	0.540, 0.491	1.648	0.1992
	*BCL11A*	CCAG	0.293	0.290, 0.296	0.025	0.8752
	*BCL11A*	ACGG	0.18	0.158, 0.204	2.439	0.1183

3	*HBS1L-MYB*	TTTATGT	0.725	0.692, 0.760	3.898	0.0484
	*HBS1L-MYB*	GTTATGT	0.145	0.139, 0.152	0.243	0.6223
	*HBS1L-MYB*	**GCCGCAC**	0.047	0.065, 0.028	5.257	0.0219^*∗*^
	*HBS1L-MYB*	TCCGCAC	0.031	0.041, 0.021	2.25	0.1336
	*HBS1L-MYB*	TTTATGC	0.018	0.023, 0.012	1.13	0.2877

Significant SNPs	*HBS1L-MYB*	TTATGT	0.87	0.830, 0.912	9.837	0.0017^*ϕ*^
	*HBS1L-MYB*	**CCGCAC**	0.078	0.106, 0.049	7.739	0.0054^*∗*^
	*HBS1L-MYB*	TTATGC	0.018	0.023, 0.012	1.15	0.2835
	*HBS1L-MYB*	**CTGCAC**	0.012	0.020, 0.003	4.181	0.0409^*∗*^

^*∗*^Significant risk haplotypes (*p* < 0.05). Order of significant SNPs: rs9376090, rs9399137, rs4895441, rs9389269, rs9402686, and rs9494142. ^*ϕ*^Haplotype associated with low HbF levels.
