# Supplementary material for: Existence of HbF Enhancer Haplotypes at HBS1L-MYB Intergenic Region in Transfusion-Dependent Saudi β-Thalassemia Patients
Source: Biomed Res Int. 2017 Feb 9;2017:1972429. doi: 10.1155/2017/1972429 (PMC5322420; doi:10.1155/2017/1972429)
Supplement: Supplementary file 1 — Table S1: Allelic association of 14 SNPs related to BCL11A, HBS1L-MYB, and HBG2 promoter region in patients with β-thalassemia and elevated HbF levels. Table S2: Frequency of haplotypes of SNPs in HBG2, BCL11A, and HBS1L-MYB compared between High HbF and Control cohorts. [file 1972429.f1.pdf]

Supplementary Table S1: Allelic association of 14 SNPs related to *BCL11A*, *HBS1L-MYB* (HMIP), and *HBG2* promoter region in patients with β-thalassemia and elevated HbF levels

SNP ID	Candidate Gene	Chromosome Position	Alleles (EA/OA)	High HbF Vs. Control			Low HbF Vs. Control	
				Odds Ratio (95% CI)	χ^2	*p* value	χ^2	*p* value
**rs2071348**	HBG2 region	11: 5242916	T/G	1.159 (0.793-1.694)	0.587	0.4434	4.531	**0.0333***
**rs7482144**	HBG2 region	11:5254939	C/T	1.145 (0.764-1.715)	0.432	0.5108	2.573	0.1087
**rs5006884**	HBG2 region	11: 5352021	T/C	1.339 (0.898-1.996)	2.06	0.1512	0.616	0.4325
**rs766432**	BCL11A	2:60492835	A/C	1.045 (0.716-1.526)	0.054	0.8161	0.223	0.6366
**rs11886868**	BCL11A	2:60493111	T/C	1.472 (1.038-2.087)	4.738	**0.0295***	0.101	0.7507
**rs4671393**	BCL11A	2:60493816	G/A	1.088 (0.742-1.596)	0.19	0.6626	0.292	0.589
**rs7557939**	BCL11A	2:60494212	A/G	1.549 (1.092-2.198)	6.057	**0.0138***	0.083	0.7735
**rs28384513**	HBS1L-MYB	6:135055071	G/T	1.212 (0.789-1.862)	0.779	0.3774	2.274	0.1315
**rs9376090**	HBS1L-MYB	6:135090090	C/T	2.343 (1.283-4.279)	8.038	**0.0046***	6.331	**0.0119***
**rs9399137**	HBS1L-MYB	6:135097880	C/T	2.152 (1.168-3.964)	6.284	**0.0122***	3.288	0.0698
**rs4895441**	HBS1L-MYB	6:135105435	G/A	2.316 (1.283-4.182)	8.116	**0.0044***	1.005	0.316
**rs9389269**	HBS1L-MYB	6:135106021	C/T	2.101 (1.180-3.742)	6.577	**0.0103***	0.725	0.3944
**rs9402686**	HBS1L-MYB	6:135106679	A/G	2.101 (1.180-3.742)	6.577	**0.0103***	0.725	0.3944
**rs9494142**	HBS1L-MYB	6:135110502	C/T	2.218 (1.288-3.819)	8.577	**0.0034***	0.418	0.518

* Significant association *p*-values ($p < 0.05$) for the allelic model. EA: effect allele tested for association; OA: Other allele; *p: p*-value unadjusted; Chromosome position as per GRCh38.p2 Assembly

Supplementary Table S2: Frequency of haplotypes of SNPs in *HBG2, BCL11A* and *HBS1L-MYB* compared between High HbF and Control cohorts.

Block	Candidate Gene	Haplotype	**High HbF Vs. Control**		**Low HbF Vs. Control**	
			χ^2	*p* **Value**	χ^2	*p* **value**
1	HBG2 region	TCC	3.451	0.0632	5.873	**0.0154***
	HBG2 region	TCT	0.605	0.4367	0.039	0.8426
	HBG2 region	**GTT**	7.959	**0.0048***	0.731	0.3925
	HBG2 region	**GTC**	7.825	**0.0052**$^{\varphi}$	0.79	0.3742
	HBG2 region	GCC	2.012	0.1561	2.353	0.125
	HBG2 region	**TTC**	4.185	**0.0408**$^{\varphi}$	0.858	0.3543
2	BCL11A	**ATGA**	5.041	**0.0247***	0.083	0.7735
	BCL11A	CCAG	0.19	0.6626	0.292	0.589
	BCL11A	**ACGG**	6.792	**0.0092**$^{\varphi}$	0.021	0.884
3	HBS1L-MYB	TTTATGT	2.884	0.0894	0.67	0.4132
	HBS1L-MYB	GTTATGT	0.114	0.7355	0.196	0.658
	HBS1L-MYB	**GCCGCAC**	4.611	**0.0318***	1.571	0.21
	HBS1L-MYB	TCCGCAC	3.811	0.0509	0.341	0.5593
	HBS1L-MYB	TTTATGC	1.023	0.3119	0.322	0.5707
Significant SNPs	HBS1L-MYB	**TTATGT**	7.258	**0.0071**$^{\varphi}$	**-**	**-**
	HBS1L-MYB	**CCGCAC**	8.763	**0.0031***	**-**	**-**
	HBS1L-MYB	TTATGC	1.023	0.3118	**-**	**-**

*Significant risk haplotypes ($p<0.05$). Order of significant SNPs: rs9376090, rs9399137, rs4895441, rs9389269, rs9402686 and rs9494142. $^{\varphi}$ Haplotype associated with low HbF levels
